# Location, but not defensive genotype, determines ectomycorrhizal community composition in Scots pine (*Pinus sylvestris* L.) seedlings

**DOI:** 10.1002/ece3.7384

**Published:** 2021-03-27

**Authors:** Jim Downie, Andy F. S. Taylor, Glenn Iason, Ben Moore, Jonathan Silvertown, Stephen Cavers, Richard Ennos

**Affiliations:** ^1^ Ashworth Laboratories Institute of Evolutionary Biology University of Edinburgh Edinburgh UK; ^2^ Centre for Ecology and Hydrology Penicuik UK; ^3^ School of Natural Sciences Bangor University Wales UK; ^4^ The James Hutton Institute Aberdeen UK; ^5^ Institute of Biological and Environmental Sciences University of Aberdeen Aberdeen UK; ^6^ Hawkesbury Institute for the Environment Western Sydney University Penrith NSW Australia

**Keywords:** community composition, ectomycorrhizal fungi, evolution, mutualism, Scots pine (*Pinus sylvestris*), secondary metabolites

## Abstract

1. For successful colonization of host roots, ectomycorrhizal (EM) fungi must overcome host defense systems, and defensive phenotypes have previously been shown to affect the community composition of EM fungi associated with hosts. Secondary metabolites, such as terpenes, form a core part of these defense systems, but it is not yet understood whether variation in these constitutive defenses can result in variation in the colonization of hosts by specific fungal species.
2. We planted seedlings from twelve maternal families of Scots pine (*Pinus sylvestris*) of known terpene genotype reciprocally in the field in each of six sites. After 3 months, we characterized the mycorrhizal fungal community of each seedling using a combination of morphological categorization and molecular barcoding, and assessed the terpene chemodiversity for a subset of the seedlings. We examined whether parental genotype or terpene chemodiversity affected the diversity or composition of a seedling's mycorrhizal community.
3. While we found that terpene chemodiversity was highly heritable, we found no evidence that parental defensive genotype or a seedling's terpene chemodiversity affected associations with EM fungi. Instead, we found that the location of seedlings, both within and among sites, was the only determinant of the diversity and makeup of EM communities.
4. These results show that while EM community composition varies within Scotland at both large and small scales, variation in constitutive defensive compounds does not determine the EM communities of closely cohabiting pine seedlings. Patchy distributions of EM fungi at small scales may render any genetic variation in associations with different species unrealizable in field conditions. The case for selection on traits mediating associations with specific fungal species may thus be overstated, at least in seedlings.

## Graphical abstract

Host defense genotype has previously been shown to affect associations with ectomycorrhizal (EM) fungi, but the extent to which secondary metabolites such as terpenes affect these associations is unknown. We conducted a reciprocal transplant field experiment to test the effect of varying terpene genotype on the EM community composition of individual seedlings and found that the only the location of a seedling affects community composition. Other processes affecting EM community composition at small scales, such as environmental preference and interspecific interactions, may thus preclude the expression of any effect of genotype in wild conditions.

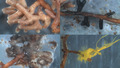

## 1 INTRODUCTION

**FIGURE 1:**
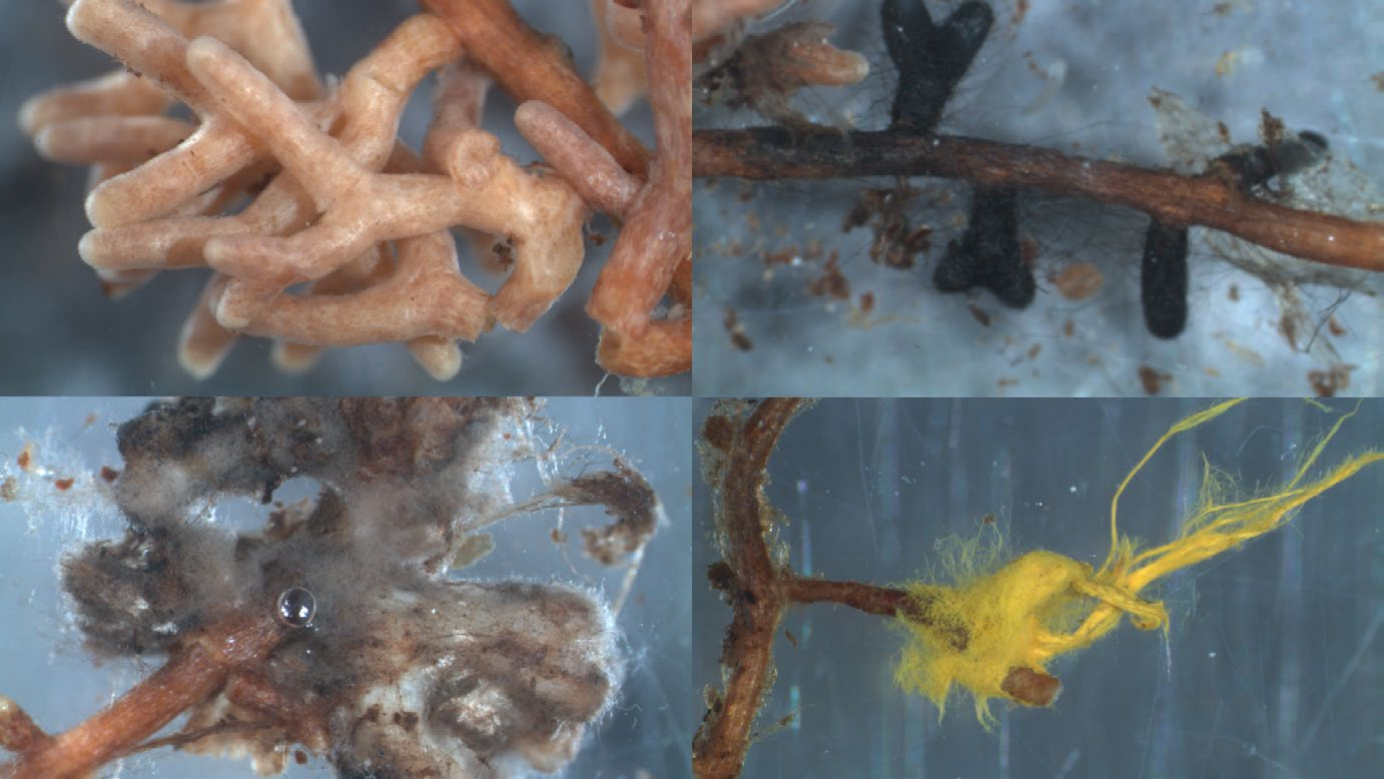
Various ectomycorrhizal root tips observed in the experiment, showing some of the various morphotypes observed. Molecular IDs (from top left, left to right, and top to bottom): 1. unidentified Cantharellales sp. 2. *Cenococcum geophilum*; 3. *Suillus variegatus*; 4. *Piloderma olivaceum*

Ectomycorrhizal (EM) fungi are important components of forest ecosystems worldwide, despite forming associations with only 2% of plant species (Brundrett & Tedersoo, 2018; Smith & Read, 2008; Tedersoo et al., 2010). There are an estimated 20,000–25,000 species worldwide (Tedersoo et al., 2010), with a diverse assortment of morphologies and functions belowground (Agerer, 2001) (Figure 1). Associations between plant hosts and EM fungi are usually mutualistic, involving an exchange of sugars and soil nutrients, but interactions can vary along a continuum, from parasitism to commensalism to mutualism (Karst et al., 2008). Such outcomes are context‐dependent, varying between different host genotypes (Hoeksema et al., 2012), fungal genotypes (Burgess et al., 1994), and on the environmental context of the interaction, such as soil nutrient availability or soil moisture levels (Hoeksema et al., 2010; Karst et al., 2008; Patterson et al., 2018). Due to this genetic influence, it has been suggested that both partners should be subject to ongoing coevolution, in order to maximize the benefit received from the interaction (Hoeksema, 2010). Evidence of variation between host genotypes in the amount of benefit received from EM fungi, with both single‐strain and multispecies inoculum, has been demonstrated for a number of traits, including relative growth rate, height, and biomass (Hoeksema et al., 2012; Hoeksema & Thompson, 2007; Pickles et al., 2015). This genetic variation can be geographically structured, as a result of local adaptation on the part of both the host and its fungal partners (Rúa et al., 2016, 2018; Thompson, 2005).

It is not yet fully understood which host traits might be under selection to regulate interactions with EM fungi (Hoeksema, 2010; Piculell et al., 2018), but a good candidate might be host defense. (Hoeksema, 2010; Hoeksema et al., 2012). In order for an EM fungus to successfully colonize a plant root, it must interact with and successfully overcome the host's defense systems (Garcia et al., 2015), and thus, individual components of a host's immune system might be under selection in order to exclude colonization by poor mutualists or parasitic fungi (Hoeksema, 2010). Variation in host defense has previously been shown in a number of studies to affect interactions between hosts and EM fungi. Tagu et al. (2005) found that a quantitative trait locus (QTL) in black cottonwood (*Populus trichocarpa*) associated with ectomycorrhiza formation was linked to other QTLs involved in the interaction with a leaf rust fungi, while Piculell et al. (2018) found that loblolly pine (*Pinus taeda*) susceptibility to two fungal pathogens affected the proportion of root tips colonized by the EM genus *Thelephora* in multiple soil sources. However, these studies examined plants grown in individual pots in glasshouse trials, with conditions that differ strongly from those found in natural environments.

In natural environments, EM fungi are patchily distributed at small scales, often less than 1 m (Bahram et al., 2016; Lilleskov et al., 2004; Pickles & Anderson, 2016; Pickles et al., 2010, 2012). This small‐scale spatial variation may render any effects of host genotype irrelevant unless the effects are large. A number of studies conducted on trees from natural environments found that spatial position strongly determined the EM community composition, with no differences in the EM community composition of different host genotypes at any position on a transect (Bubner et al., 2013; Lang et al., 2013; Saari et al., 2005). However, field experiments conducted on trees varying in defensive characteristics have still shown effects of genotype on EM community composition (Gehring et al., 2014; Lamit et al., 2016). In a common garden of 15‐year‐old clonally replicated narrowleaf cottonwood (*Populus angustifolia*) trees, host genotype explained 13% of the variation in EM associations (Lamit et al., 2016), and these EM communities have been shown to covary with communities of fungal leaf pathogens (Lamit et al., 2015). Similarly, a field experiment conducted on pinyon pine (*Pinus edulis*), looking at two co‐occurring genotypes of pinyon pine (*P. edulis*) which varied in their resistance to a stem‐boring moth, showed that moth‐resistant trees had much lower percentages of ascomycete colonization (Gehring et al., 2014; Sthultz et al., 2009). This finding was replicated in a common garden experiment, which also demonstrated a twofold difference in the Shannon diversity index of fungal communities between the two genotypes (Patterson et al., 2018). Together, these results suggest that variation in host defense may be a strong candidate for investigation for effects on EM associations, with effects replicable both in the field and experimentally.

One important defense system in trees, particularly conifers, are secondary metabolites. These diverse compounds are produced in shoots, needles, and roots (Hiltunen, 1976) and have been shown to deter grazing both by invertebrates and vertebrates, including deer (e.g., Iason et al., 2011; O'Reilly‐Wapstra et al., 2007), and can also alter the composition of nearby vegetation (Iason et al., 2005). There have been indications that these defensive compounds may be involved in interactions with EM fungi; for example, phenolic compounds have been shown to accumulate in root tips of known incompatible host–fungi pairings (Malajczuk et al., 1982; Molina & Trappe, 1982; Voigt et al., 2000), suggesting a role of secondary metabolites in mediating successful colonization. In pines, terpenes are one of the primary groups of defensive compounds (Mumm & Hilker, 2006). It has been shown that terpenes have antifungal effects in vitro (Eckhardt et al., 2009; Marei et al., 2012; Zhang et al., 2016 *)* and that these effects are active against both saprotrophic and EM fungi via exposure both to pure vapors and leaf litter (Ludley et al., 2008; Melin & Krupa, 1971). Curiously, EM fungi were found to be more strongly growth inhibited than saprotrophic fungi (Ludley et al., 2008). However, it is not yet known whether variation in constitutive terpenes within a host can affect the colonization of hosts by different groups of fungi.

The native Scots pine (*Pinus sylvestris*) forests of Scotland provide a good system within which to test the effects of terpenes on EM fungal colonization (Downie et al., 2020). These pinewoods have a well‐documented EM community, which varies in response to the dominant rainfall gradient in Scotland (Jarvis et al., 2013, 2015). Additionally, seedlings from different populations have been shown to be genetically differentiated in their response to EM inoculation (Downie et al., 2020). Scots pine populations also show a geographic bias in their terpene chemotype; much of this chemotypic variation is captured in the variation between trees in their issue concentration of Δ^3^‐carene (Forrest, 1980; Thoss et al., 2007), which is usually the second‐most abundant terpene in Scots pine (O'Reilly‐Wapstra et al., 2007). Trees from the northwest regional populations typically have a higher frequency of zero Δ^3^‐carene chemotypes, although there is much intrapopulation variability, and the presence/absence of Δ^3^‐carene has been shown to affect multiple ecological interactions of Scots pine, such as deterring grazing by deer and slugs (Iason et al., 2011), the use of Scots pine trees by wood ants, and soil mite species richness (Iason et al., 2012).

To determine whether constitutive terpenes affect the association of host plants with different EM fungi in the field, a reciprocal field transplant was set up to investigate whether pine defensive genotype or terpene character affects the selection of mycorrhizal partners by pine seedlings growing in the field. Seedlings from six populations were grown reciprocally in grids in each of the six originating forests in a fully factorial cross. Mycorrhizal communities were assessed through morphotyping and ITS barcoding, and terpene character through gas chromatography.

We sought to answer the following questions:

1. Does the diversity of a seedling's mycorrhizal community depend on its defensive genotype or terpene character?
2. Does the frequency of associations with different species of EM fungi vary between defensive genotypes or with terpene character?

## 2 METHODS

**FIGURE 2:**
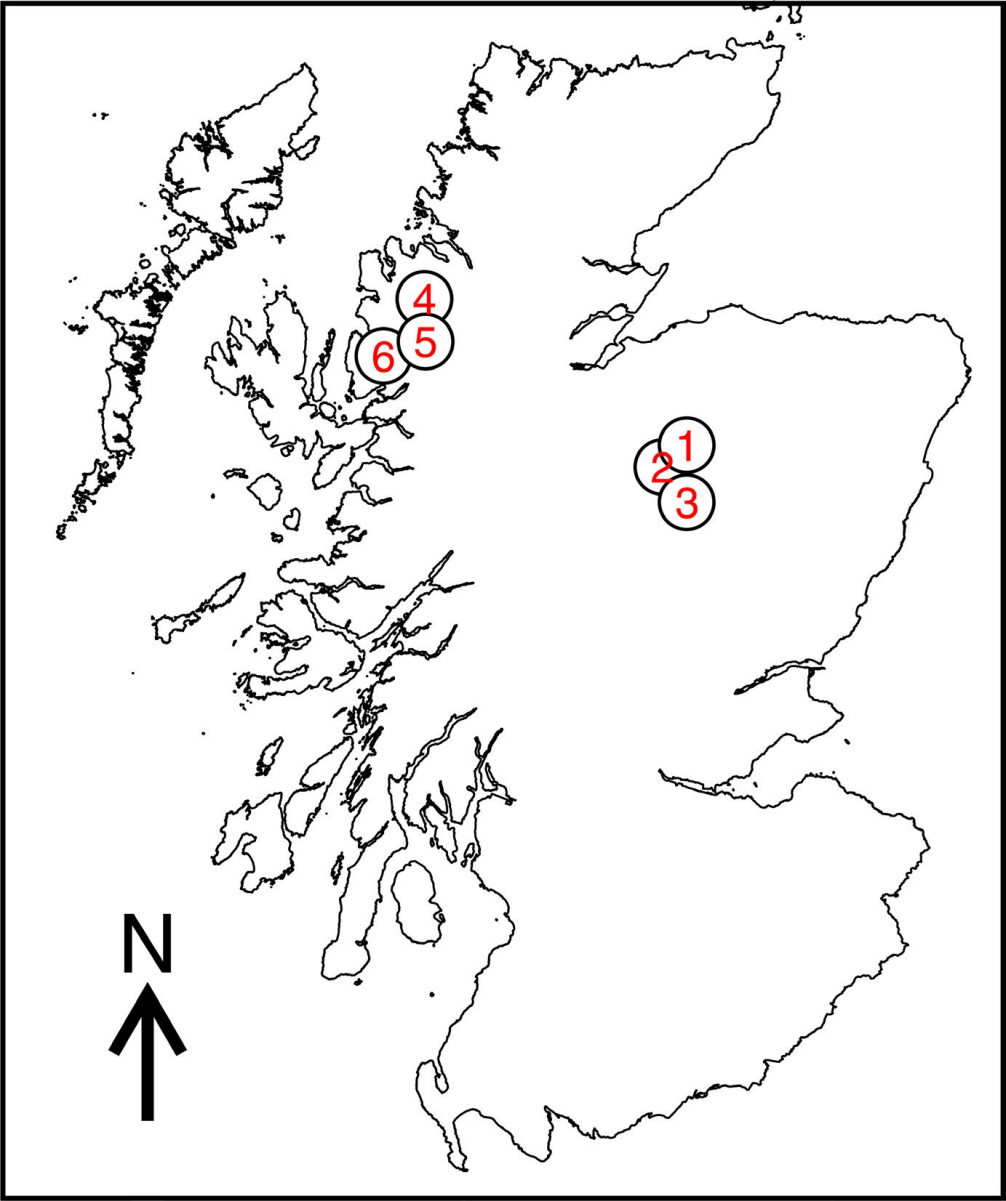
Map showing the locations of the six populations used in the study. Labels: 1 = Abernethy; 2 = Rothiemurchus; 3 = Glen Derry; 4 = Beinn Eighe; 5 = Loch Clare; 6 = Shieldaig

Cones were collected from six stands of Caledonian pinewoods in Scotland: in the west, Beinn Eighe, Loch Clair and Shieldaig, and in the east, Abernethy, Rothiemurchus, and Glen Derry (Figure 2, Table 1). Seed from each of these stands represented a population of origin, with all stands having trees with high and low Δ^3^‐carene chemotypes. Cones were selected from two mother trees at each site, with one maternal tree having a known high Δ^3^‐carene chemotype and one with a low chemotype. The Δ^3^‐carene chemotypes of the mother trees were established in previous work in this system (Iason et al., 2012). Seed from each mother tree thus represented a maternal family of half‐sib progeny, for a total of 12 maternal families across all populations.

**TABLE 1:** Coordinates of each population used in the trial, as well as climatic variables (Met Office) and soil types (Soil Information For Scottish Soils, James Hutton Institute)

Population	Code	Latitude	Longitude	Altitude (m)	MAP (mm)	MJT (°C)	Soil type
Abernethy	AB	57.21	−3.61	362	1,060.7	13.1	Humus‐iron podzol
Beinn Eighe	BE	57.63	−5.35	21	2,476.1	14.5	Peaty gleyed podzol
Glen Derry	GD	57.03	−3.58	443	1,080.4	11.7	Peaty gleyed podzol
Loch Clair	LC	57.56	−5.36	112	2,888.2	14	Peaty gleyed podzol
Rothiemurchus	RM	57.15	−3.77	307	1,060	13.5	Humus‐iron podzol
Shieldaig	*SD*	57.5	−5.64	68	2,449.7	13.1	Brown earth

Abbreviations: MAP, mean annual precipitation; MJT, mean July temperature.

Seeds were imbibed in sterilized deionized H_2_O overnight, surface sterilized in 30% H_2_O_2_, and then rinsed with deionized H_2_O, before being sown on seed trays to germinate. The seed trays were filled with a 1:7 mixture of peat to vermiculite which had been sterilized by autoclaving. Following germination but before transplanting, the root systems were checked to ensure there were no visible signs of EM colonization. Seedlings were then planted in each of the six stands, with each planting stand representing a site. At each site, three grids were set up at chosen locations (between 100 m and 2 km apart). Each grid was set up in a patch of moss, outwith the vicinity of any nonpine EM host plants, to try and reduce spatial heterogeneity. Grids were 50 × 50 cm in total extent, with 5‐cm intervals on both axes. This grid size was chosen as it was found in preliminary work (A.F.S. Taylor, pers. comm.) to reduce spatial heterogeneity in soil conditions and EM community composition between seedlings while remaining far apart enough that the root systems would not overlap during the growing period. Between 3 and 5 seedlings per maternal family were transplanted in each grid at randomly selected intersections. Seedlings were planted into the upper organic layers of the soil (FH horizon), where the majority of EM fungal diversity is found (Baier et al., 2006; Dahlberg, 2001; Read & Perez‐Moreno, 2003; Rosling et al., 2003). After 3 months of growth in the field, seedlings were harvested, and root systems were separated from the shoots. Both were stored in bags at −20°C for later analysis.

### 2.1 Community characterization

The EM fungal communities on the roots of each seedling were characterized by morphotyping. Root tips were observed under a stereo microscope, and assigned to morphotypes based on their appearance, with identification where possible (Agerer, 2001). The number of root tips for each morphotype on each seedling was counted, with a tip being considered as each individual end where a single colonization point branched (i.e., a bifurcating root tip was considered as two tips). Tuberculate mycorrhizas were counted as one tip. We also counted the number of non‐EM root tips. For each morphotype on a seedling, 1–5 root tips were stored in Eppendorf tubes and frozen at −20°C for identification using molecular barcoding of the ITS region.

For each morphotype from each grid, a minimum number of five tips from different seedlings were selected for sequencing where possible. For all morphotypes from the three eastern sites, we extracted DNA from root tips using the Qiagen DNEasy Plant Mini Kit, with the following modification to the protocol: At the final elution stage, DNA was eluted from the spin column in 2 × 25 μl steps. We performed PCR for each extract using the fungal‐specific primers ITS4 (5′‐TCCTCCGCTTATTGATATGC‐3‐) and ITS1‐*F* (5′‐CTTGGTCATTTAGAGGAAGTAA‐3′). These primers are capable of amplifying DNA from both Ascomycetes and Basidiomycetes, although each primer shows a small amplification bias toward one or the other (Bellemain et al., 2010). PCR was performed in a 25 μl reaction mix with the following makeup: 10.5 μl H_2_O, 5 μl 10X PCR buffer, 1 μl each of ITS4 and ITS1‐F at 20 μM concentration, 4 μl MgCl_2_ at 50 μM, 1 μl dNTPs at 20 μM, 1 μl BSA at 20 mg/ml, 0.5 μl BioTaq Polymerase, and 1 μl template DNA. The following settings were used on the thermocycler: 95°C for 5 min, then 30 cycles of 94°C for 30 s, 55°C for 30 s, and 72°C for a minute, before at 72°C for 10 min and holding at 10°C. Samples which yielded a single band (>99% of samples) following PCR were then sent for forward Sanger sequencing at Eurofins GATC using primer ITS1‐F. Cleanup of the PCR reactions was performed at this facility.

For morphotypes from the three western sites, a different protocol for extraction and PCR was used, as the previous protocol did not reliably produce amplifiable DNA. DNA was extracted using the Sigma‐Aldrich REDExtract‐n‐Amp Plant Kit, with the following protocol modifications: Individual root tips were placed into wells in a 96‐well plate with 10 μl of the extraction solution and incubated in a thermocycler at 65°C for 10 min, then 95°C for 10 min. After this, 30 μl of dilution solution was added to each extract. PCR was performed using the accompanying REDExtract PCR mix, in 20 μl reaction mix with the following makeup: 10 μl REDE mix, 8.6 μl H_2_O, 0.4 μl each of ITS1‐F and ITS4 at 20 μM, and 1 μl of template DNA. The following program was used on the thermocycler: 94°C for 1 min, followed by 30 cycles of 94°C for 1 min, 51°C for 1 min, and 72°C for 1 min. Following these cycles, samples were held at 72°C for 10 min before being held indefinitely at 10°C. PCRs were then cleaned up using Sigma‐Aldrich illustra ExoProStar 1‐Step, which was diluted 1 in 4 with PCR‐grade H_2_O. DNA concentrations of each sample were quantified on a gel, diluted to recommended concentrations, and samples which yielded a single band (>99% of samples) were forward Sanger sequenced at Edinburgh Genomics using primer ITS1‐F. The use of this alternative extraction and PCR protocol is unlikely to have affected the results, as the difference in efficacy meant that we simply had to conduct less repeat extractions to obtain all the sequences required.

Following sequencing, chromatograms from both sequencing sets were basecalled using the ABI KB Basecaller (version 1.4.1; Hyman et al. (2010)), and sequences were trimmed at each end to remove low‐quality regions with Phred scores lower than 20. Sequences were not manually edited to remove unsure base calls, in order to increase reproducibility. These sequences were used for all downstream analyses. For taxonomic identification of morphotypes, only sequences over 50 bp in length were retained, as this length was found to allow for the identification of a small number of morphotypes which would otherwise have lacked identification. Filtered sequences were searched against the UNITE fungal taxonomy database (Nilsson et al., 2019) using BLAST+ (Camacho et al., 2009). Only sequences known to be from EM fungal lineages were used for further analysis (Tedersoo et al., 2010). For each morphotype, we manually assigned an identity based on the identities of the tips for which sequence data were available. Morphotypes were assigned a species‐level identification if at least one sequence was >99% similar to a reference sequence, or genus‐level identification if above 95%. In cases where morphotypes were already putatively identified to genus or species, this information was additionally considered when assigning identity.

**FIGURE A1:**
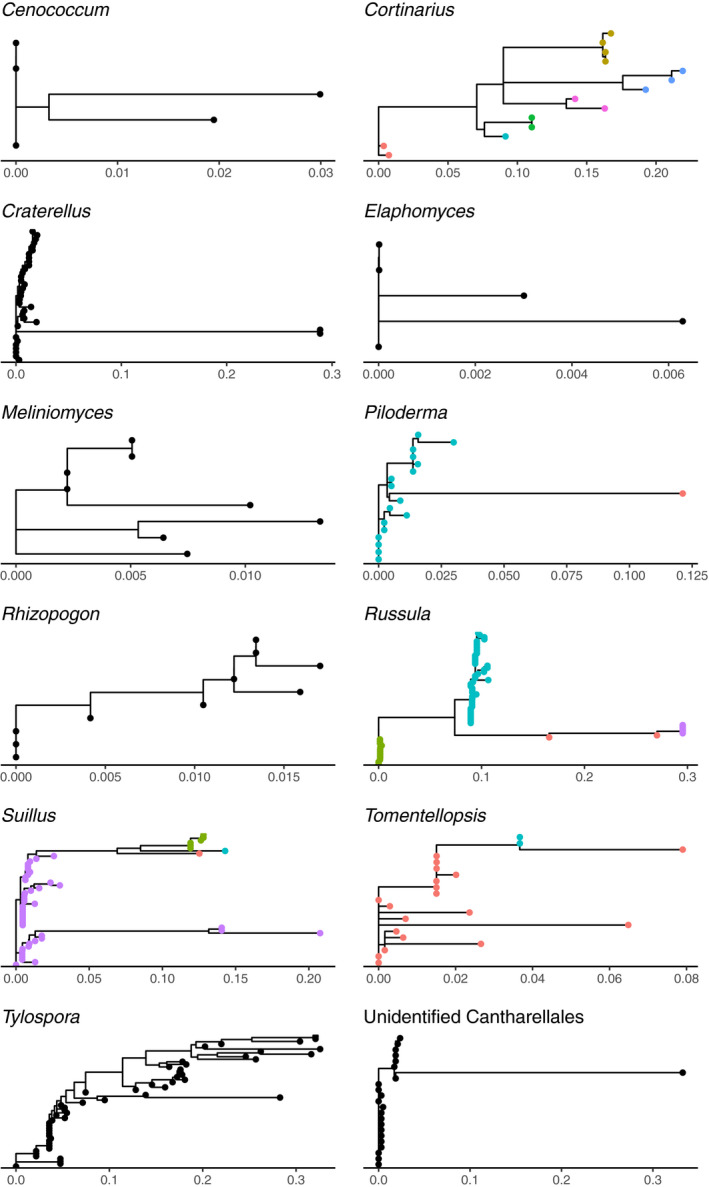
Maximum‐likelihood trees of sequences for each genus, showing consistent taxonomic identification of sequences. Where tips are colored, colors represent different species as identified by BLAST

To ensure consistency of morphological categorization, where morphotypes had sequences suggesting multiple species, taxonomic IDs were then rechecked against microscope images of each morphotype to ensure that they had been correctly grouped and split where appropriate according to morphological features. To check the consistency of taxonomic identification, sequences for each genus were aligned in *mafft* (Katoh & Standley, 2013). These alignments were then used to construct rapid bootstrap maximum‐likelihood trees in *RAxML* (Stamatakis (2014); trees in Figure A1), using the GTRGAMMA model and 1,000 bootstrap iterations. These trees were used to determine whether sequences assigned only to genus should be considered as a single taxon, or whether multiple taxa were present within each genus. In three cases, we merged taxa based on poor taxonomic resolution: *Tomentellopsis* was considered as a single taxon due to a poorly resolved tree, a clade of *Russula* containing two putatively identified species (*Russula caerulea* and *Russula sardonia*) was merged to a single taxon, *Russula* sp., and *Tylospora* was treated as a single taxon despite cryptic genetic diversity apparent in the tree due to an inability to morphologically distinguish clades which had been counted as a single morphotype (Figure A1).

### 2.2 Gas chromatography and terpene data

The presence or absence of Δ^3^‐carene within Scots pine is strongly genetically determined by a single locus, with homozygotes with the Δ^3^‐carene allele having high Δ^3^‐carene proportions, heterozygotes showing intermediate levels, and seedlings without any copy of the allele producing no Δ^3^‐carene (Hanover, 1966; Kinloch et al., 1986; Pohjola et al., 1989). Because of this, it cannot be assumed that the Δ^3^‐carene chemotypes of seedlings would match that of their maternal tree, as the paternal tree was not identified. To account for this, terpenes were extracted from needle tissue to assess the terpene character of each seedling. We assumed that needles would have a similar chemotype to roots, as terpenes in these tissues have previously been shown to be correlated in mature trees (Hiltunen, 1976; Napierała‐Filipiak et al., 2002).

At the end of the experiment, seedlings were collected into small zip‐lock bags onto ice and then stored at −20°C until analysis. Needles from seedlings for four planting sites (Beinn Eighe, Loch Clair, Shieldaig, and Rothiemurchus) representing all maternal families were sampled for terpene characterization. Terpenes were extracted from finely chopped, small needle samples (mean = 36 mg) in duplicate in 500 μl of n‐hexane containing 54.5 ug/ml of isobutylbenzene as an internal standard. GC‐FID was carried out using a 30 m RT BetaDEXsm chiral column with an internal diameter of 0.25 mm and a film thickness of 0.25 μm (Thames, Restek), operated with helium as a carrier gas at a flow rate of 1 ml/min. One microliter of extract was injected into a split/splitless inlet operating at 180°C with a split ratio of 40 and an initial oven temperature of 60°C. The initial temperature was held for 2 min before increasing at a rate of 3.5°C/min to 130°C and then at 10°C/min to 210°C. The FID was operated at 250°C. Mass spectrometer readings were transformed to measurements of μg terpene/g needle dry mass.

In total, there were 16 terpenes (in order of decreasing proportional abundance: α‐pinene, Δ^3^‐carene, terpinolene, β‐pinene, β‐caryophyllene, myrcene, limonene, an unknown terpene, germacrene‐D‐4‐ol, camphene, α‐humulene, cis‐β‐ocimene, γ‐terpinene, germacrene‐D, bicyclogermacrene, tricyclene). For each seedling, each pair of measurements for each terpene was averaged, and then, the proportional concentration of each terpene for each seedling was calculated, considering pairs of enantiomers as single terpenes. Using these proportional concentrations, we calculated the Shannon diversity index (−∑*c_i_*log(*c_i_*)), where *c_i_* is the proportional concentration of terpene (*i*), to characterize the chemical diversity of each seedling. To determine what factors affected terpene character, we used a linear mixed model to determine whether there were effects of parental chemotype, western or eastern seed origin, or location of growth on a seedling's chemical diversity (Equation 1). Family was included as a random effect to control for nonindependence between seedlings within a family. Parental chemotype was a binary factor (high or low), and population of origin and site were factors with the same six levels.

(1) Chemodiversity∼Parental Chemotype+Population+Site+random(Family)

### 2.3 Statistical analysis

All statistics were carried out in R version 4.0 (R Core Team, 2019). At the end of the experiment, it was found that 127 of the 673 surviving seedlings remained uncolonized. Of the seedlings for which terpene data were gathered, 283 of the 343 seedlings were colonized. These seedlings were kept in the datasets for all analyses except where specified, as these seedlings may have failed to be colonized due to their genotype or terpene character. To check that all species present at each site was recovered, species accumulation curves were calculated using the specaccum function in *vegan* (Oksanen et al., 2020), using the “exact” method, and the first‐order jackknife model.

To investigate how EM community composition varied across all samples, we conducted an ordination using NMDS with the metaMDS function in the *vegan* package (Oksanen et al., 2020), with the uncolonized seedlings removed. Species occurring on only one seedling were also removed to allow for easier convergence. The number of axes (*k*) was selected by comparing the stress of ordinations with *k* = 1–8, and selecting *k* such that the stress was <0.1 and not decreasing by large amounts. Following this method, *k* was chosen to be 4. For each of the 12 maternal families at each site, we estimated the mean and standard error on each ordination axis.

**FIGURE 3:**
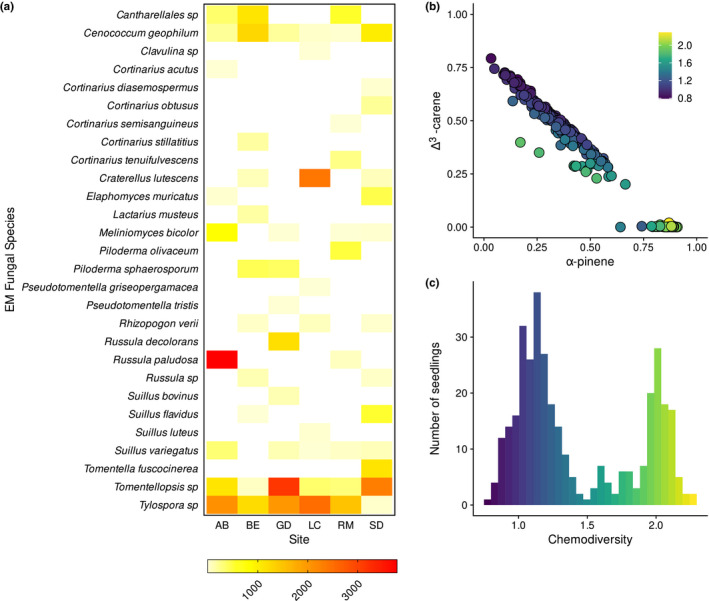
Summary graphs: EM species abundance and the terpene composition of individual seedlings. (a) Heatmap showing the total number of root tips of each EM species found at each of the six sites. (b) Plot of proportional α‐pinene concentration against proportional Δ^3^‐carene concentration, showing a strong linear decrease in Δ^3^‐carene concentration with increasing α‐pinene concentration. Colors indicate increasing terpene chemodiversity. (c) Histogram of terpene chemodiversity, showing two distinct peaks, indicating high/low Δ^3^‐carene phenotypes

For all models involving terpenes, we used the smaller dataset of 343 seedlings for which there was terpene character data. Terpene character was included in models as the chemical diversity of terpenes within a seedling. This value was found to correlate strongly with other potential measures of terpene character, including the proportional concentration of 3‐carene (*r* ^2^ = 0.84) (Figure 3b).

### 2.4 Richness and diversity analyses

To determine whether the species richness or true diversity (Jost, 2006) of the fungal community on a seedling varied between sites, families, or with terpene character, we used GLMMs fitted in *glmmTMB* in R (Brooks et al., 2017). Because true diversity is measured in species richness, we assumed that seedlings with 0 species richness also had zero true diversity. Models investigating family effects used the full dataset, with the formula in Equation 2, but models investigating terpene character used the smaller terpene dataset, with the formula in Equation 3. For species richness data, we modeled the response using a Poisson error structure. The total number of root tips was included in the models to account for root system size, as larger root systems are likely to be colonized by more species, and grid (nested within site) was included as a random effect to control for variation within sites. Effects of site, maternal family, and terpene character were examined using likelihood ratio tests between models with these effects removed. Post hoc comparisons for differences between sites were conducted using the *emmeans* package in R, with Tukey HSD corrections applied (Lenth, 2021).

(2) Diversity index∼Total tips+Site+random(Grid)+random(Family)

(3) Diversity index∼Total tips+Chemodiversity+Site+random(Grid)

### 2.5 Turnover analyses

To determine whether community composition varied among genotypes or sites, we modified a method used by Shutt et al. (2020) to model turnover in community composition of OTUs (β‐diversity) for any combination of explanatory variables of interest. This method uses a Bayesian GLMM framework, with the number of root tips of a given fungal species on a given individual seedling as the response variable, with the log total number of root tips on a seedling used as an offset to control for the number of opportunities for colonization. With this model structure, variation in the abundance of different fungal species (OTUs) across many factors of interest can be tested using random effects (Equation 4). The random effect OTU tests for overall variation in the abundance of different species, while the random effects such as Site:OTU and Family:OTU test for variation in the abundance of different fungal species among levels of these factors, showing turnover in community composition within variables. These random effects can be composed of both categorical effects and covariates (such as chemodiversity). The contribution to total variance of each of these random effects can then be calculated, allowing partitioning of variance among all variables of interest. We constructed these models in *MCMCglmm* in R (Hadfield, 2010), using parameter‐expanded priors for all random effects. Models were run for 2,000,000 iterations each, with the first 40,000 discarded as burn‐in and thinning conducted every 200 iterations. Traces of the posteriors were checked visually to ensure good mixing and convergence, and effective sample sizes of the posteriors were checked to ensure they were at least approximately 200.

(4) $$\begin{array}{c} \text{Number of root tips}∼\log(\text{Total root tips})+\text{random}(\text{OTU})\\ \;\;+\text{random}(\text{Site}:\text{OTU})+\text{random}(\text{Grid}:\text{OTU})+\text{random}(\text{Family}:\text{OTU}) \end{array}$$

In particular, we conducted two sets of models, one estimating the effect of maternal family using the full dataset and one estimating the effect of terpene character using the reduced dataset. In the first case, models were fitted with only an intercept as a fixed effect, with species (OTU) fitted as a random effect, as well as random effects to test for turnover in community composition among sites (Site:OTU), grids (Grid:OTU), and families (Family:OTU) (Equation 4). For the terpene models, we fitted chemodiversity as a fixed covariate and tested for turnover in EM community composition using a random slope for each species in response to chemodiversity. We also included random effects to test for variation in overall species abundance (OTU), as well as turnover in community composition among sites (Site:OTU) and grids (Grid:OTU) (Equation 5).

(5) $$\begin{array}{c} \text{Number of root tips}∼\log(\text{Total root tips})+\text{Chemodiversity}\\ \;\;+\text{random}(\text{OTU})+\text{random}(\text{Chemodiversity}):\text{OTU}\\ \;\;+\text{random}(\text{Site}:\text{OTU})+\text{random}(\text{Grid}:\text{OTU}) \end{array}$$

## 3 RESULTS

**FIGURE A2:**
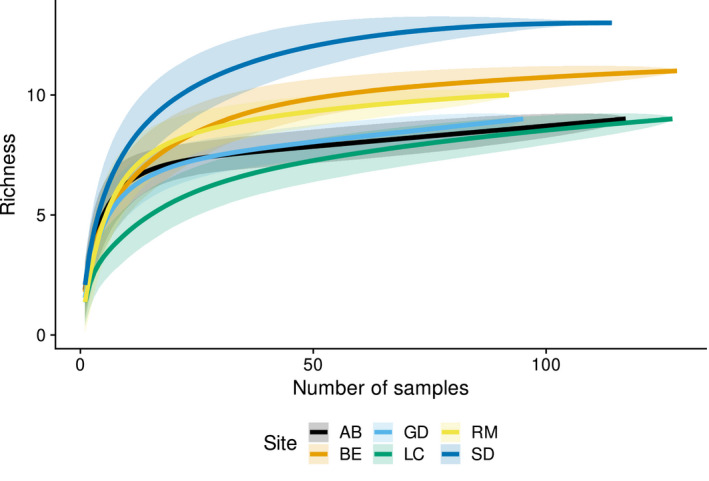
Species accumulation curves for each of the six sites, showing that most fungal species present in the grids at each site were recovered

In total, 673 seedlings survived to the end of the experiment, of which, 546 were colonized by EM fungi during the growing period of 3 months. Uncolonized seedlings typically had small root systems with little to no branching, suggesting poor overall seedling success. We counted approximately 44,400 root tips in total, assigning them to 76 morphotypes. We assigned these morphotypes to 28 EM taxa, with a total of 16 genera and 28 species (Figure 3a). Of these, five were singletons, occurring on only a single seedling: *Pseudotomentella tristis*, *Pseudotomentella griseopergamacea*, *Cortinarius semisanguineus*, *Cortinarius acutus*, and *Suillus luteus*. Species accumulation curves showed that at each site, all species present within each grid were recovered (Figure A2). On average, there were 10.2 ± 0.65 (SE) EM species per site, and 5.6 ± 0.31 (SE) species per grid. However, on average individual seedlings only associated with 2.1 ± 0.04 (SE) EM species. Data on terpene character was collected for 349 seedlings from four of the six sites, for which 282 were colonized.

**FIGURE 4:**
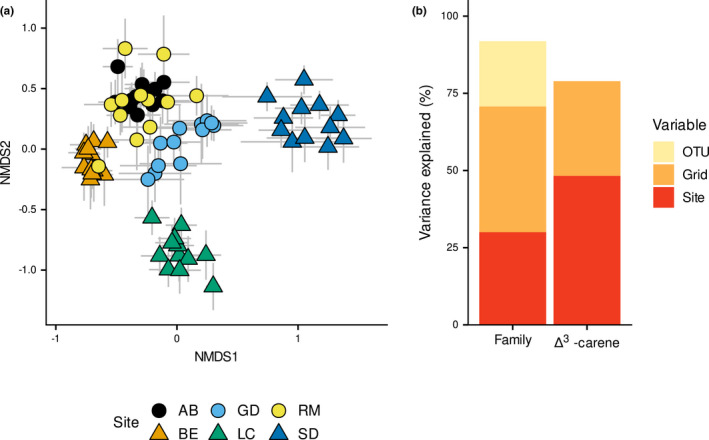
Variation in EM fungal community composition between sites and genotypes. (a) NMDS ordination of seedling EM communities showing variation between sites. Each point represents the mean NMDS score ± SE for one of the 12 maternal families at a particular site. Circles represent eastern sites, and triangles represent western sites. Plots of all other axes are available in Figure A3. (b) Percentage variance explained by each component in the maternal family turnover model (Equation 4) and Δ^3^‐carene turnover model (Equation 5), calculated from the estimates of the posterior modes. Site = turnover between sites, Grid = turnover between grids, OTU = unexplained variation in the abundance of fungal species. Genotype effects are not shown in the legend as no effects were found

Following the analysis of stress for different dimensions, the number of dimensions selected for the NMDS was four, with a stress of 0.09. The NMDS ordination showed that the community composition of EM fungi varied between sites, with communities from Loch Clare and Shieldaig being particularly distinct when viewed on axes 1 and 2 (Figure 4a). The other sites were broadly similar in community composition, particularly Abernethy and Rothiemurchus, which are geographically very close. Broadly speaking, western sites (Beinn Eighe, Shieldaig, and Loch Clare) were more distinct from one another, while all three eastern sites (Abernethy, Rothiemurchus, and Glen Derry) showed strong degrees of overlap. Within each site, maternal families did not appear to differ strongly from one another, with error bars commonly overlapping for most pairs of maternal families.

On average, seedlings had a mean chemical diversity of 1.43 ± 0.02 (SE). However, chemical diversity was strongly bimodally distributed (Figure 3a), with seedlings lacking Δ^3^‐carene having a mean chemodiversity of 1.98 (±0.02 SE), and seedlings with Δ^3^‐carene present having a mean chemodiversity of 1.15 (±0.02 SE). This bimodal distribution was driven strongly by the proportional increase of α‐pinene in seedlings lacking Δ^3^‐carene (Figure 3b).

We found that terpene chemical diversity was strongly genetically determined, with seedlings from western seed sources or from maternal trees with a low Δ^3^‐carene chemotype having higher chemical diversity (low Δ^3^‐carene mother tree: 0.39, SE = 0.07, *z* = 5.8, *p* < 0.001; Western seedlings: 0.24, SE = 0.07, *z* = 3.5, *p* < 0.001). By comparison, we found no effect of seedling location, considered as an effect of site (Site: Chisq = 0.27, *df* = 1, *p* = 0.61).

### 3.1 Richness and diversity analyses

On average, seedlings associated with 1.7 fungal species (SE = 0.04). We found no effect of terpene chemodiversity or family on the species richness of a seedling's EM community (Terpene chemodiversity: Chisq = 2.7, *df* = 1, *p* = 0.1; Maternal family: Chisq = 0.47, *df* = 1, *p* = 0.49), but did find that richness varied among sites in the full dataset, but not significantly in the smaller terpene dataset (full dataset: Chisq = 13.9, *df* = 5, *p* = 0.017; terpene dataset: Chisq = 7.14, *df* = 3, *p* = 0.07). Post hoc analysis using *emmeans* found pairwise differences between a number of site pairs in both models: In the full dataset, Shieldaig had higher richness than Loch Clare (*t* = −3.5, *p* = 0.08) and Glen Derry (*t* = −3.3, *p* = 0.013), while in the terpene dataset, the only significant difference was between Beinn Eighe and Loch Clare (*t* = 2.7, *p* = 0.04).

The mean diversity of a seedling's EM community was lower than the species richness (1.4 effective species, SE = 0.03). We found a difference among sites in diversity of seedling's fungal community (full dataset: Chisq = 19.7, *df* = 5, *p* = 0.001; terpene dataset: Chisq = 10.6, *df* = 3, *p* = 0.014), but no effect of terpene chemodiversity (Chisq = 0.6, *df* = 1, *p* = 0.42) or maternal family on diversity (Chisq = 3.7, *df* = 1, *p* = 0.053), as in the richness model. Similar to the richness models, post hoc analysis using *emmeans* showed pairwise differences between a number of site pairs in both models: In the full dataset, Shieldaig had higher diversity than Rothiemurchus (*t* = −3.6, *p* = 0.004), Loch Clare (*t* = −4.2, *p* = 0.0005), and Glen Derry (*t* = −4.8, *p* < 0.0001), and similarly Beinn Eighe had a higher richness than Loch Clare (*t* = 3.0, *p* = 0.03) and Glen Derry (*t* = 3.7, *p* = 0.004). In the terpene dataset, the only difference found was again between Beinn Eighe and Loch Clare (*t* = 3.5, *p* = 0.003).

### 3.2 Turnover analyses

We found no evidence that seedling genotype, considered as maternal families, had an effect on which EM species associated with a given seedling (*σ* ^2^ attributable to maternal family = 0.13, 95% CI: ~0 to 0.31). We found strong evidence for significant variation in EM species abundance between each of the six sites (*σ* ^2^ attributable to site = 25.0, 95% CI: 9.7 to 43.9), and strong evidence for variation within sites at the grid level (*σ* ^2^ attributable to Grid = 30.5, 95% CI: 19.0 to 44.0). There was also evidence for variation in the overall abundance of different EM species (*σ* ^2^ attributable to OTU = 21.4, 95% CI: 6.5 to 41.1). In total, the residual variance was 5.8 (95% CI: 5.3 to 6.5). This means that turnover due to spatial variation (site and grid) accounted for 71% of the variance in the model (Figure 4b).

For the smaller terpene dataset, we found no evidence that terpene character affected EM community composition (Slope of chemodiversity: 0.36, 95% CI: −0.1 to 0.8; *σ* ^2^ attributable to Chemodiversity:OTU slope = 0.26, 95% CI: ~0 to 0.9). However, we still found evidence for variation between sites (*σ* ^2^ attributable to site: 43.2, 95% CI: 11.0 to 83.9), as well as variation between grids (*σ* ^2^ attributable to Grid = 19.9, 95% CI: 10.1 to 31.7). There was no evidence for the overall variation in the abundance of different EM species (*σ* ^2^ attributable to OTU = 16.5, 95% CI: ~0 to 39.6). In total, the residual variance was 6.1 (95% CI: 5.2 to 7.0).

## 4 DISCUSSION

Overall, we found strong evidence for turnover in EM fungal community composition between sites, but found no evidence that maternal families associated differently with fungal species. We also found no evidence that maternal families varied in the richness or diversity of their associated mycorrhizal communities. Similarly, there was no evidence that terpene chemodiversity affected the probability of association with different fungal species, nor the richness or diversity of a seedling's EM community.

As previous work in vitro on terpenes has suggested that EM and saprotrophic fungi may respond to specific terpenes (Ludley et al., 2008; Ludley et al., 2009; Melin & Krupa, 1971), the lack of effect found here may reflect a small effect size compared with the small‐ and large‐scale spatial parameters investigated here. Δ^3^‐carene prevalence varies among trees within stands (Iason et al., 2012), and all trees release terpenes into the environment as volatiles from roots and shoots, as well as through leaf litter (Ludley et al., 2008; Ludley, et al., 2009), affecting a much larger area than the grids in this study. The effects of these environmental terpenes on EM fungi are still unknown, but they have been shown to affect the diversity of ground vegetation in these pine systems (Iason et al., 2005), as well as nitrogen mineralization rates by soil microbiota (White, 1991). Thus, it is plausible that terpenes may still affect EM community composition in Scots pine, but studies at broader scales that compare zones of influence between mature trees of differing Δ^3^‐carene genotype may be required to find them.

While we found no effect of plant defensive genotype, we did find that the spatial location of a seedling, both among and within sites, was the sole determinant of the EM fungal community on individual seedlings (Figure 4b). EM community composition within Scotland has previously been shown to vary in response to the prevailing east–west rainfall gradient, as well as with altitude and increased nitrogen deposition (Jarvis et al., 2013), and such effects have been demonstrated more broadly in other studies across Europe (Cox et al., 2010; van der Linde et al., 2018; Suz et al., 2014). Although we did not test for effects of environmental variables directly in this study, it is likely that the variation in EM community composition among sites found in this study is a result of such environmental variation.

At smaller, within‐site scales, EM community composition has been shown within Scotland to vary in response to small‐scale variation in soil conditions, such as soil temperature, organic matter content, and pH (Jarvis et al., 2015). Furthermore, stochasticity in fungal dispersal, as well as competition among different fungal genets, leads to patchy fungal distributions at small scales, with patch sizes varying from tens of cm to meters (Anderson et al., 2001; Bahram et al., 2016; Beiler et al., 2010; Guidot et al., 2001; Lilleskov et al., 2004; Pickles & Anderson, 2016; Pickles et al., 2010, 2012; Sawyer et al., 1999). The strong effect of grids within site found in this study likely reflects this small‐scale heterogeneity in EM fungal distributions. It is likely that given the small number of grids at each site, and this small‐scale spatial variation in EM community composition, we did not effectively capture the full EM fungal diversity at each site. However, the in‐depth sampling of each grid using many seedlings allowed us to robustly investigate the effect of host defensive genotype with many replicates within these spatially homogeneous areas.

A number of previous field studies investigating the effect of host genotype on EM community composition have found similar results highlighting the importance of spatial position over genotype. In a study along a transect in a stand of common beech (*Fagus sylvatica*), EM community composition was found to vary spatially, but different host genotypes at each sample point were not found to differ in their EM associations (Bubner et al., 2013). Similar results were found in an experiment by Lang et al. (2013) exploring the EM communities at transect points between three individual common beech trees. A study on five individual Scots pine (*P. sylvestris*) trees in a native stand in Scotland also found no differences in EM associations between host genotypes (Saari et al., 2005). Studies like these, conducted on closely related trees growing in proximity to one another, may thus not provide much insight into whether genotypes vary in their interactions with EM fungi.

In these studies, genotypes were not hypothesized, a priori, to have an effect due to differences in a key trait. By comparison, a glasshouse study conducted on fast‐ and slow‐growing clones of Norway spruce (*Picea abies*), Korkama et al. (2006) found that these clones differed in their EM fungal communities, both in terms of diversity and composition. Similarly, in a pair of common garden and field studies from a single stand of pinyon pine (*P. edulis*), two genotypes of tree varied in their canopy architecture as well as in their resistance to a stem‐boring moth. These genotypes were shown to vary in their proportion of ascomycete to basidiomycete EM fungi, as well as the associated diversity of their EM communities (Gehring et al., 2014; Patterson et al., 2018; Sthultz et al., 2009). Thus, even in field conditions, host genotype effects may be discoverable if genotypes are selected to explore the effects of specific traits. Interestingly, the genotypes also appeared to exhibit different growth rates in the common garden trial, with moth‐susceptible genotypes exhibiting increased shoot growth compared to moth‐resistant genotypes, which may reflect different nutritional requirements and hence EM associations (Patterson et al., 2018). In the experiment we report here, the two genotypes from each population came from a high 3‐carene mother and a low 3‐carene mother, which we hypothesized might affect the EM associations of a seedling. The lack of evidence found for an effect of co‐occurring host defensive genotypes from multiple populations thus allows us to robustly conclude that the proportion of Δ^3^‐carene in needles has no effect on host‐associated EM communities.

The lack of genetic signature in EM associations found here also highlights the potential importance of the life history of trees in shaping the evolutionary response to EM fungi (Batstone et al., 2018; Downie et al., 2020). High density‐dependent mortality due to competition among seedlings for light and space may mean that selectivity, through association only with “high quality” EM partners, may impose significant competitive penalties via opportunity costs. Furthermore, if the presence or absence of a species at a particular location is unpredictable, or the quality of particular EM species can vary, for example, due to temporal or spatial heterogeneity in soil resources, host generalism may be important for survival, precluding adaptation (Batstone et al., 2018). This generalism is likely to be more pronounced in long‐lived species, such as trees, where shifting environmental conditions may change the composition of EM communities, disrupting any adaptation that has taken place. In agreement with this, a previous study in this system found no evidence of local adaptation of Scots pine seedlings to EM fungi (Downie et al., 2020), and overall evidence for local adaptation in EM interactions is lacking (Rúa et al., 2018). Instead, host genotype effects on EM community composition found in other studies (Korkama et al., 2006; Patterson et al., 2018) may reflect differing nutritional requirements due to variation in growth rate, with hosts promoting associations that best meet their needs through preferential allocation (Bever, 2015), rather than reflecting evolution for increased or decreased association with specific fungal species.

Finally, the lack of genetic effects on EM associations found here underscores the importance of conducting experiments on host genotype in more realistic field conditions as well as in the glasshouse. Glasshouse experiments are powerful tools that can disentangle effects that cannot be manipulated in the field, and are useful in understanding traits underlying specific effects (Englund & Cooper, 2003; Forero et al., 2019; Limpens et al., 2012). However, they typically remove all factors other than those of interest, with the potential to exaggerate those effects (Forero et al., 2019). Thus, it is important that future experiments looking for differences among host genotypes in mycorrhizal interactions incorporate more realistic conditions, particularly if there is an interest determining whether there is potential for selection to act on these traits in natural environments. Combining mechanistic insight from glasshouse trials with practical implications from these field experiments will provide deeper insights into the potential for evolution in these systems.

## 5 CONCLUSIONS

Using a reciprocal transplant experiment, we found that host defensive genotype, considered as both maternal family and terpene chemodiversity, had no effect on the community composition of EM fungi on Scots pine seedlings. Instead, spatial location, considered as differences among and within sites, was the sole factor determining EM community composition. These results reflect the well‐described understood ecology of EM fungi at regional and local scales within Scotland and highlight the potential importance of host life history in determining evolutionary responses to mycorrhizal fungi. Future work seeking to understand evolutionary responses to EM fungi should seek to work across hosts of different life stages, as well as use field experiments to incorporate potentially confounding effects of EM fungal ecology.

## CONFLICT OF INTEREST

The authors have no conflicts of interest to declare.

## AUTHOR CONTRIBUTIONS

**Jim Downie:** Data curation (lead); formal analysis (lead); investigation (equal); methodology (equal); project administration (equal); resources (equal); visualization (lead); writing – original draft (lead). **Andy F. S. Taylor:** Conceptualization (lead); investigation (equal); methodology (equal); project administration (equal); resources (equal); supervision (equal); writing – review and editing (equal). **Glenn Iason:** Conceptualization (lead); funding acquisition (lead); investigation (equal); methodology (equal); resources (equal); supervision (equal); writing – review and editing (equal). **Ben Moore:** Data curation (supporting); investigation (equal); methodology (equal); writing – review and editing (equal). **Jonathan Silvertown:** Formal analysis (supporting); resources (equal); supervision (equal); writing – review and editing (equal). **Stephen Cavers:** Formal analysis (supporting); resources (equal); supervision (equal); writing – review and editing (equal). **Richard Ennos:** Formal analysis (supporting); supervision (equal); writing – review and editing (equal).

## Figures and Tables

**FIGURE A3:**
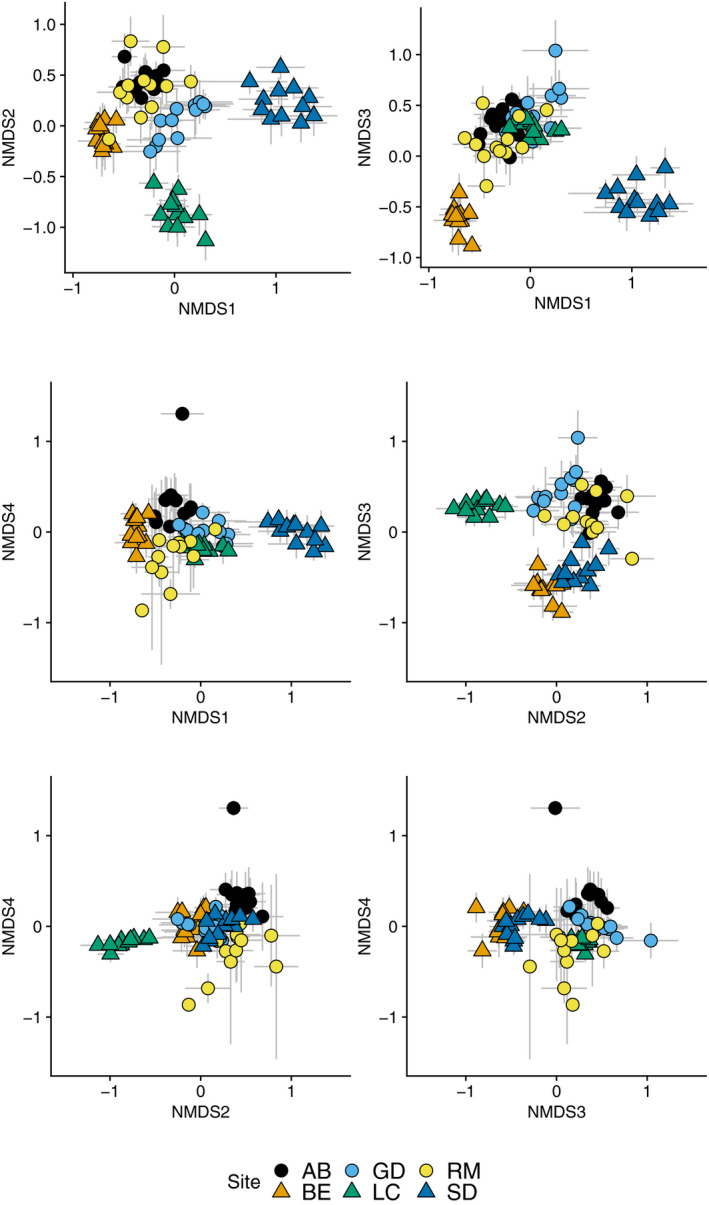
The full NMDS ordination with all four calculated axes plotted against each other. Each point represents the mean NMDS score ± SE for one of the 12 maternal families at a particular site. Circles = eastern sites, Triangles = western sites

## Data Availability

The sequences used to determine species identity are available on Genbank (Accession numbers MW004337–MW004543). The data used in this paper, scripts containing the models, and files containing the turnover model runs are available on DataDryad (https://doi.org/10.5061/dryad.ncjsxksth).
